# Orbitomaxillofacial Mucormycosis Requiring Complex Multifactorial Management

**DOI:** 10.1097/GOX.0000000000001927

**Published:** 2018-10-02

**Authors:** Anna K. Steve, Valerie A. Hurdle, Jevon Y. Brown

**Affiliations:** From the Section of Plastic Surgery, Department of Surgery, University of Calgary, Calgary, Alberta.

## Abstract

Mucormycosis is a rare fungal infection caused by ubiquitous fungi in the order Mucorales. It is the most rapidly progressing fulminant fungal infection that mimics necrotizing soft-tissue infections. Overwhelming fungal sepsis develops quickly and mortality rates approach 70%. Culture negative necrotizing infections and cutaneous necrosis following a vascular pattern should raise suspicion for this rare entity. We describe avoiding mortality in a case of orbitomaxillofacial mucormycosis multifactorially treated with: radical serial debridement, topical amphotericin B irrigation and dressings, parenteral amphotericin B, and hyperbaric oxygen therapy. Tissue biopsy was central to confirming the diagnosis and directing multimodal management that ultimately prevented dissemination to the central nervous system and mortality.

Cutaneous mucormycosis is a rare necrotizing fungal infection, with immunocompromised patients at increased risk.^1,2^ Clinical presentation of Mucor resembles necrotizing soft-tissue infections or other etiologies with compromised soft-tissue vascularity. Mucor may present as either a primary cutaneous process or hematogenous spread from a deep source. In primary cutaneous cases, lesions begin as papules that quickly develop ulceration, vascular invasion, and thrombosis of underlying vessels.^1,2^ Hematogenous dissemination progresses rapidly, with mortality rates approaching 70%.^3^

Culture negative necrotizing infections and cutaneous necrosis following a vascular pattern should raise suspicion for mucormycosis. Bacterial and fungal stains are often negative, and diagnosis is confirmed with tissue biopsy positive for branching, nonseptate fungal hyphae on microscopy^1^ or fungal polymerase chain reaction. Aggressive tissue debridement and intravenous amphotericin B are the mainstay of treatment, but mortality remains high. Adjunct treatment with hyperbaric oxygen,^4^ and topical amphotericin B have demonstrated positive results.^5,6^

A case of successful treatment of orbitomaxillofacial mucormycosis is described. Although delayed diagnosis resulted in considerable morbidity; mortality was avoided with a combination of radical surgical debridement, systemic and topical amphotericin B, and hyperbaric oxygen therapy.

## CASE PRESENTATION

**Fig. 1.**
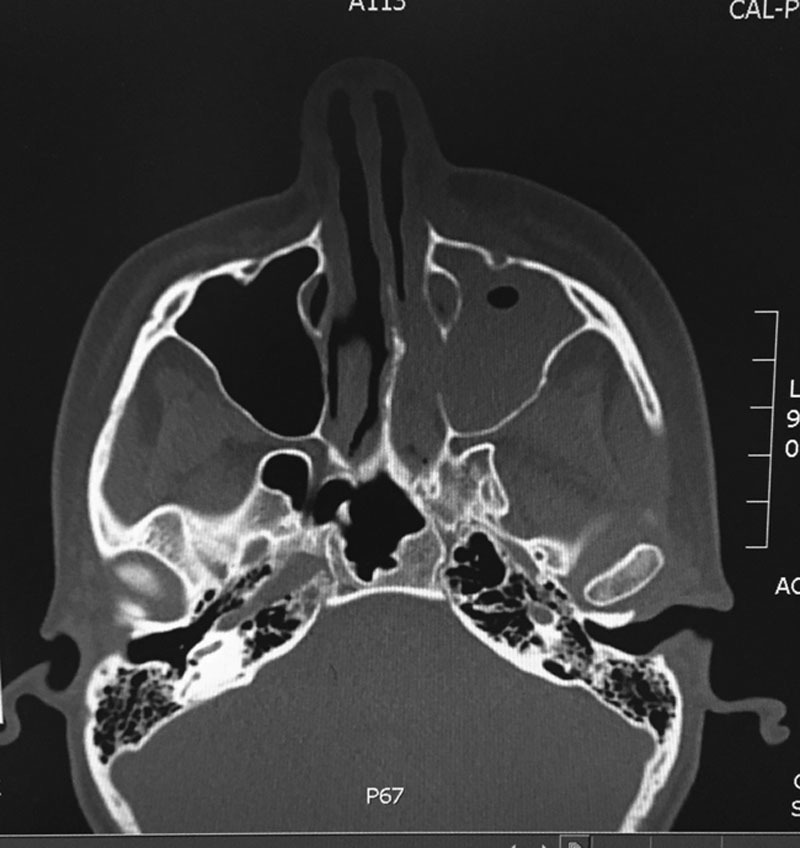
Axial view of computed tomography images demonstrating initial maxillary sinusitis.

A 25-year-old female with poorly controlled diabetes mellitus was referred urgently to plastic surgery for possible necrotizing soft-tissue infection of her left orbitomaxillary region. Seven days prior, the patient had been admitted to hospital for a suspected bacterial sinusitis (Fig. 1) and diabetic ketoacidosis. Following stabilization, the patient was discharged on oral doxycycline. The patient returned to hospital with 2 days of worsening left facial pain, left facial droop, profound edema, and grayish discoloration to the skin to the left orbital region.

At time of plastic surgery assessment, all vital signs were within normal limits, with no evidence of fever. Marked left facial edema, erythema, and an area of grayish skin involving the left face and ear were present. Cranial nerve examination demonstrated a left-sided unilateral facial nerve palsy and lack of left trigeminal nerve sensation (V1-2) The left neck was tender, with palpable cervical adenopathy. Left ear examination revealed a thickened erythematous tympanic membrane with fluid in the middle ear. There were no signs of nasal mycosis or invasive fungal rhinosinusitis on nasal endoscopy. Aside from preseptal cellulitis and facial nerve paralysis, left ocular examination revealed no abnormalities in vision or extraocular motion. Contralateral head and neck examination was within normal limits. Laboratory investigations revealed leukocytosis (13.7 × 10^9^/L, elevated blood glucose (20.3 mmol/L) and diabetic ketoacidosis. Computed tomography scan showed nonspecific subcutaneous tissue stranding with no subcutaneous gas or localized collections. Maxillary sinusitis was improving, when compared with imaging from her prior admission.

**Fig. 2.**
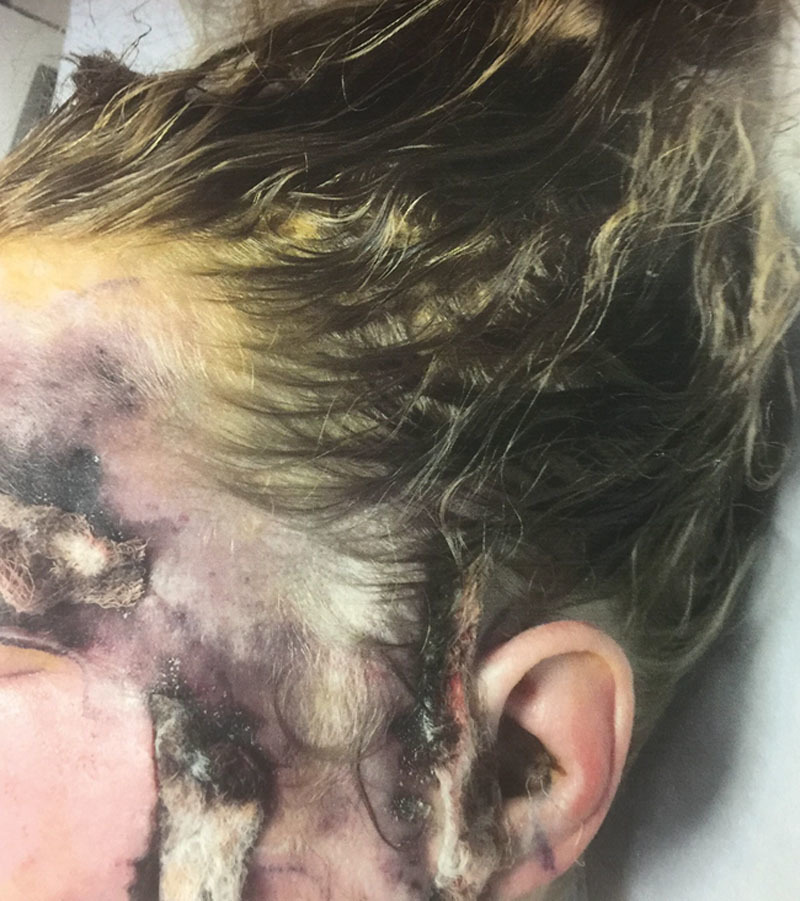
Twenty-four hours after initial exploration, demonstrating the area of progressing necrosis

The patient was transferred to the operating room for urgent surgical exploration by the plastic surgery team. Thrombosis of the superficial temporal and supraorbital arteries with dry necrosis in the vascular territory supplied by these vessels was present (Fig. 2). Intraoperative cultures for anaerobic and aerobic bacteria, acid-fast bacilli, and fungal organisms all demonstrated no growth.

**Fig. 3.**
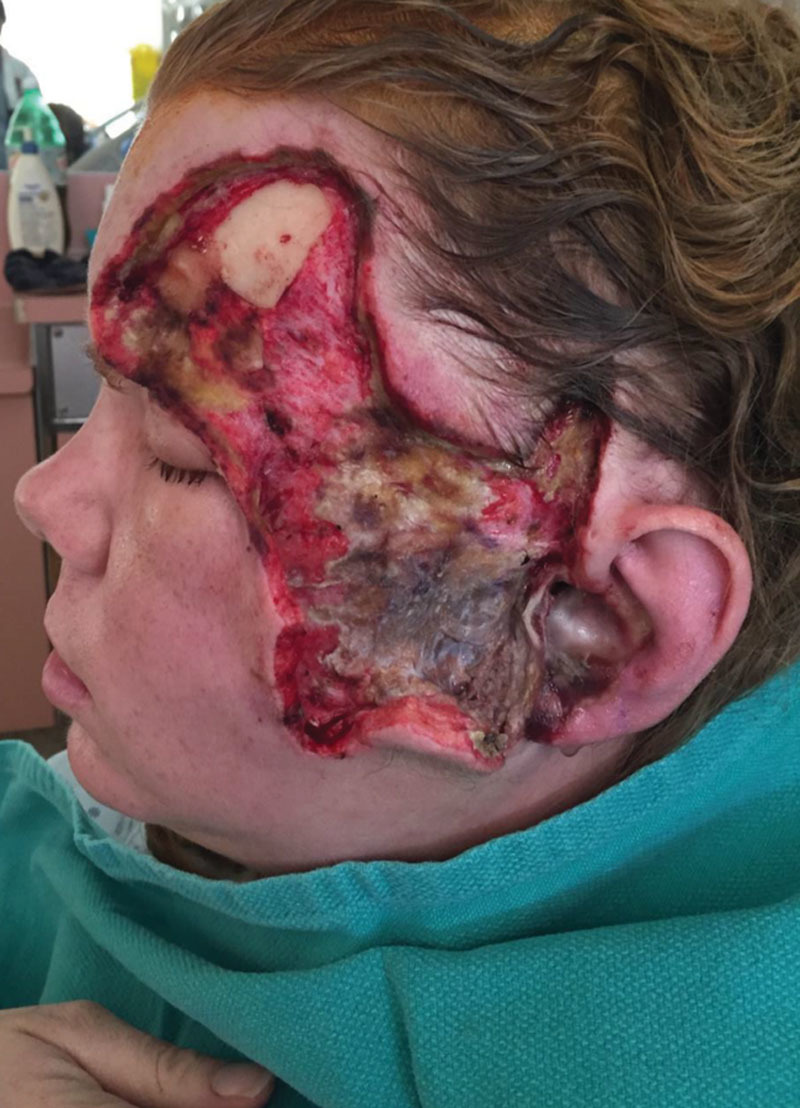
Wound following 2 previous debridements with gray, necrotic tissue overlying the parotid and ear demonstrating continued disease progression.

The patient returned to the operating room for 11 debridements for progressing left facial necrosis (Fig. 3) over the following 6 weeks. The extent of the debridement required collaboration with an otolaryngologist and ophthalmologist for 2 of these debridements. Numerous repeat cultures were negative, with eventual diagnosis being made on tissue biopsy, positive for fungal hyphae, and polymerase chain reaction, confirming a diagnosis of orbitomaxillofacial mucormycosis with Rhizopus oryzae approximately 2 weeks after presentation.

Due to continued tissue necrosis adjunct measures were implemented: the wound was irrigated with amphotericin B deoxylate (0.5 mg/ml) intraoperatively and dressed with wet to dry dressings soaked with amphotericin B deoxylate (0.5 mg/ml) 4 times daily, with exclusion of the exposed middle ear. Intravenous amphotericin B liposomal injection at 10 mg/kg was used for systemic rhinomucormycosis coverage. Additionally, hyperbaric oxygen therapy was instituted. A total of 30 dives at 2.5 atmospheres for 2.5 hours each were planned. However, hyperbaric oxygen therapy was discontinued after 3 sessions due to left sided mandibular pain and patient intolerance.

**Fig. 4.**
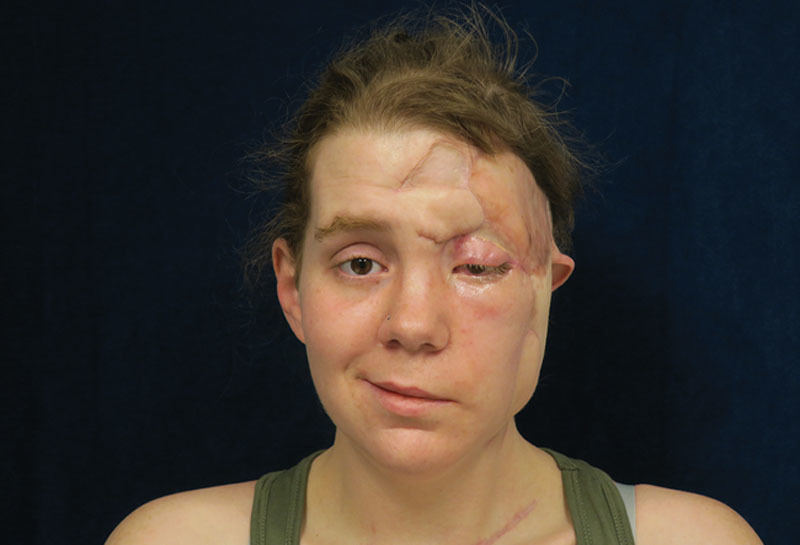
Soft-tissue reconstruction, 5 months postoperatively from ALT flap. A small area of forehead wound breakdown required subsequent closure with a pedicled forehead flap, also shown here.

Vascular thrombosis progressed to involve the entire left external carotid artery and all terminal branches. Final soft-tissue deficit included the left middle ear (tympanic membrane and ossicles), large portion of the external ear, temporal bone and facial nerve, parotid gland, mastoid sinus, maxillary sinus, ethmoid sinus, sphenoid sinus, upper eyelid and brow, facial muscles, superior oblique muscle, trochlea, overlying facial muscles, subcutaneous tissue and skin. Hospital stay was complicated by acute renal failure leading to anuria and volume overload that required hemodialysis, and ongoing volatile glucose control. The patient was managed on the Burn Unit with assistance from infectious disease, nephrology, and internal medicine. Reconstruction with an anterolateral thigh flap and skin graft was used to cover the large remaining soft-tissue deficit, with a plan for secondary facial reanimation, bone anchored hearing device, and levator reconstruction (Fig. 4). Due to the extensive soft-tissue involvement, the descending branch of the lateral circumflex femoral artery and vein were anastomosed to the superior thyroid artery and vein at the recipient site. A full-thickness skin graft was used to cover a portion of the superior eyelid.

## DISCUSSION

There is little information published about mucormycosis in plastic surgery literature.^1,7^ Due to it’s tendency to mimic other necrotizing conditions, plastic surgeons may be involved in care of patients with this rare entity. Dry, nonpurulent soft-tissue necrosis in a vascular distribution should alert surgeons to consider a diagnosis of mucormycosis, even before negative bacterial and fungal cultures results. High suspicion for mucormycosis diagnosis should prompt tissue biopsy to be inspected for fungal hyphae.^1^ Increased knowledge of this rare condition will allow plastic surgeons to consider diagnosis early, as negative bacterial and fungal stains delay diagnosis leading to potentially fatal treatment delays. Intraoperative irrigation with amphotericin B and amphotericin B soaked dressings and hyperbaric oxygen were used in addition to conventional management by the plastic surgery team with success in this case.

Cutaneous mucormycosis is rare condition, usually affecting immunocompromised hosts. As it mimics other causes of soft-tissue necrosis, plastic surgeons should be familiar with the diagnosis and management of this rare entity. Early diagnosis allows for prompt, targeted treatment, preventing potentially fatal delays in treatment. The mainstays of treatment are radical debridement and systemic antifungal therapy. Augmenting these main interventions with topical amphotericin B irrigation and dressings and hyperbaric oxygen may improve mortality rates among patient with mucormycosis.
